# WHAT CAN OTHER DEMENTIA CARE SETTINGS LEARN FROM GREEN CARE FARMS? AN EXPLORATION OF THEIR FUNCTIONS AND FORMS

**DOI:** 10.1093/geroni/igad104.0768

**Published:** 2023-12-21

**Authors:** Katharina Rosteius, Bram de Boer, Gijs Steinmann, Hilde Verbeek

**Affiliations:** Maastricht University, Maastricht, Limburg, Netherlands; Maastricht University, Maastricht, Limburg, Netherlands; Maastricht University, Maastricht, Limburg, Netherlands; Maastricht University, Maastricht, Limburg, Netherlands

## Abstract

Green Care Farms (GCFs) are an innovative long-term care alternative in dementia. They aim to better meet residents’ needs by integrating nature and animals into daily life. This study explores the functions of GCFs and possible forms of realizing them in practice. A multiple case study using ethnographic methodology was conducted on two sites: one original GCF established for seven years and one regular nursing home redesigned and recently opened aiming to apply GCF characteristics. Participatory observations (n=58), interviews (n=64), a focus group, an environmental assessment and expert panels were conducted. Six functions of GCFs were identified, crucial for the successful execution of their innovative vision in practice: providing opportunities for purposeful activities, stimulating residents, creating a community, sharing responsibilities, integrating the vision in all actions and continuously transforming. These functions may take several forms, which could be implemented in regular care, taking into account the specific local context.

